# Physical activity and body-weight regulation

**DOI:** 10.1093/ajcn/nqz132

**Published:** 2019-07-03

**Authors:** Klaas R Westerterp

**Affiliations:** NUTRIM, Maastricht University Medical Centre, Maastricht, Netherlands

Activity-induced energy expenditure, the most variable component of human day-to-day energy expenditure, is likely to respond to energy restriction or overfeeding as an adaptation to maintain energy balance. Indeed, the most extensive energy restriction study, the Minnesota experiment, showed that 58% of an energy restriction–induced reduction of energy expenditure was accounted for by a reduction of activity-induced energy expenditure, mainly caused by a reduction of body movement (1). The other 2 components of energy expenditure, maintenance expenditure and diet-induced expenditure, explained 32% and 10% of the reduction, respectively. However, overfeeding, in contrast, does not seem to induce an adaptive increase in activity-induced energy expenditure. Most overfeeding studies to date have shown no effect of overfeeding on activity-induced energy expenditure other than an increase in the cost of moving a larger body mass (2). Modern life has freed humans from weight-imposed constraints associated with a hunter-gatherer lifestyle, including predation and high-intensity physical activity. Thus, there is little selection pressure to cap weight gain, at least in the short term (3). The elegant overfeeding study by Johannsen et al. (4) in the current issue of the Journal suggests that variation in physical activity still could play a role, however, in long-term body weight regulation.

The design of the current overfeeding study has many similarities with an earlier overfeeding study by Diaz et al. (5) in which 9 young men consumed 50% above their baseline requirements for 6 wk. In the Johannsen et al. (4) study, 6 women and 29 men of similar age consumed 40% above their baseline requirements for 8 wk. The resulting excess energy intake in the 2 studies (intake during overfeeding minus baseline requirements) was 260 MJ and 269 MJ, respectively. The overfeeding-induced change in body composition was very similar as well. Subjects in the study by Diaz et al. (5) gained 7.6 ± 1.5 kg, of which 4.6 ± 1.9 kg was body fat. Subjects in the study by Johannsen et al. (4) gained 7.5 ± 1.9 kg, of which 4.2 ± 1.4 kg was body fat. Assuming an energy equivalent of 4.7 MJ/kg fat-free mass and 39.6 MJ/kg fat mass gained, 75% (Diaz study) and 66% (Johannsen study) of the excess energy was stored.

The similar results of the 2 studies, not surprisingly, resulted in similar conclusions as well. Diaz et al. concluded there was no evidence for any active overfeeding-induced energy-dissipating mechanisms. The difference between excess energy intake and energy stored was explained by increased maintenance metabolism for the larger body, increased diet-induced energy expenditure proportional to the higher intake, and increased activity-induced energy expenditure for moving the larger body mass. Johannsen et al. (4) concluded that metabolic adaptation during an energy surplus does not occur as it does in the metabolic adaptation to energy restriction.

A difference between the 2 overfeeding studies is in the follow-up measurements of changes in body composition after the overfeeding period, on a free diet without structured interventions. Diaz et al. (5) observed a weight loss of 3.9 ± 1.2 kg and a fat mass loss of 2.8 ± 1.8 kg, over 6 wk after overfeeding. Johannsen et al. (4) observed a weight loss of 4.3 ± 3.5 kg and a fat mass loss of 2.0 ± 2.7 kg, over 6 mo after overfeeding. Surprisingly, weight loss and fat loss were very similar whether they were measured at 6 wk or 6 mo after overfeeding, and were about half the overfeeding-induced gains in body mass and fat mass. Apparently, half of the overfeeding-induced gain in body fat was lost within 6 wk. The resistance to further loss of overfeeding-induced fat gain, still present at 6 mo after overfeeding, shows the consequence of overeating for weight maintenance.

A new observation in the study by Johannsen et al. (4) was that those who had a greater than predicted daily energy expenditure after overfeeding, that is, those that were more physically active, lost more fat during the follow-up period independently of total weight or fat gain with overfeeding. Diaz et al. (5) observed an increase in the energy cost of activity after overfeeding-induced weight gain. There was an increase in the energy cost of weight-bearing activity (stepping) of 11% and of non–weight-bearing activity (cycling) of 9%, in line with a 10% increase in body weight. Activities get more demanding with a higher body weight, and thus more physically active participants lost more of the gained fat during the follow-up period. Similarly, weight loss induced by energy restriction allows increasing body movement afterwards and thus greater physical activity prevents regain after weight loss (6). In addition, the Johannsen et al. study investigated the possible roles of circulating hormones and mitochondrial efficiency of skeletal muscle, mechanistic outcomes that were not studied in the earlier study.

Most importantly, neither overfeeding study provided evidence of an adaptive increase in activity-induced energy expenditure. The average physical activity level (PAL; defined as total daily energy expenditure divided by resting energy expenditure, to adjust for variation in body size) was the same at baseline and during overfeeding, respectively, 1.8 ± 0.3 and 1.8 ± 0.3 in the study by Johannsen et al. (4), and 1.8 ± 0.2 and 1.8 ± 0.2 in the study by Diaz et al. (5). Both studies included participants with a sedentary lifestyle at baseline, indicated by a PAL-value ≤1.5, and participants with a vigorously active lifestyle, indicated by a PAL-value ≥2.0. However, sedentary participants did not show an adaptive increase in PAL during overfeeding.

In conclusion, overeating is an important risk factor for long-term weight gain. Higher activity-induced energy expenditure, through a higher habitual PAL, facilitates weight recovery after intake-induced weight gain. The result is in line with an observation on long-term weight maintenance in a physically active subject, despite a 3-kg seasonal variation in body mass (7). When metabolic adaptation during an energy surplus does not occur, studies on body weight regulation can focus on prevention of overeating through increasing control of food intake. Overeating is mainly due to increased brain reactivity related to food reward. Recent findings have shown the potential of neuromodulation to reduce this food reward–related brain reactivity in individuals vulnerable to overeating (8).
